# Peripheral spondyloarthritis: a neglected entity—state of the art

**DOI:** 10.1136/rmdopen-2019-001136

**Published:** 2020-05-06

**Authors:** Philippe Carron, Ann-Sophie De Craemer, Filip Van den Bosch

**Affiliations:** 1 Rheumatology, University Hospital Ghent, Ghent, Belgium; 2 VIB Center for Inflammation Research, Ghent, Belgium

**Keywords:** Spondyloarthritis, Anti-TNF, Magnetic resonance imaging, Rheumatoid arthritis, Ankylosing spondylitis

## Abstract

Peripheral spondyloarthritis (pSpA) refers to a number of seemingly different spondyloarthritis subsets in which psoriatic arthritis (PsA) is the most common, and symptoms of arthritis, enthesitis or dactylitis predominate the clinical presentation. Although formal classification criteria for pSpA have been introduced in 2011, only a minority of epidemiological and clinical studies addressed this clinical entity as a separate disease. Moreover, research on outcome measures and treatment modalities in pSpA has been mainly focused on PsA. Subsequently, all biological treatments are off-label in patients with non-psoriatic pSpA. Its neglected status has important implications for clinical practice since the emerging group of early-diagnosed non-psoriatic pSpA patients remains poorly characterised and lacks specific treatment recommendations. This review summarises what is currently known regarding pSpA in terms of epidemiology, clinical presentation, diagnosis and therapeutic approach.

## UNIFYING CONCEPT OF SPONDYLOARTHRITIS

The spondyloarthritides (SpAs) are a heterogeneous family of inflammatory musculoskeletal disorders that share common clinical features, genetic susceptibility and pathophysiological mechanisms. Depending on the predominant clinical manifestation, SpA can be subdivided into axial SpA (axSpA), primarily affecting the axial skeleton—that is, the spine and the sacroiliac joints (SIJ), and peripheral SpA, of which the clinical presentation is determined by arthritis, enthesitis and/or dactylitis. Besides these musculoskeletal symptoms, SpA patients frequently show extra musculoskeletal manifestations (EMMs), such as acute anterior uveitis (AAU), psoriasis or inflammatory bowel disease (IBD).^1^

Originally, SpA was a generic term that referred to a set of distinct diseases: ankylosing spondylitis (AS), psoriatic arthritis (PsA), reactive arthritis (ReA) and arthritis/spondylitis associated with IBD.^2^ Around one decade ago, the Assessment of SpondyloArthritis International Society (ASAS) substituted this phenotypical approach, also known as the ‘SpA concept’, by a more comprehensive classification system for axSpA and peripheral spondyloarthritis (pSpA).^3 4^ Initially, both sets of criteria strictly separated SpA patients (ie, no possible overlap within the same patient), which rather neglected the considerable number of axSpA patients with significant peripheral involvement (25.4–51.3%, variance mainly due to varying definition of enthesitis).^5 6^ The classification of SpA patients has, therefore, gradually evolved towards an approach in which the *predominant* symptomatology determines the ASAS classification. Following this argument, patients with isolated axSpA (without peripheral involvement) or pSpA (without axial involvement) are nowadays differentiated from a third group, fulfilling both axSpA and pSpA classification criteria. In other words, it serves both clinical and research practice to no longer consider axial involvement as an ‘exclusion criterion’ for pSpA classification. It is of importance to mention that the concept of SpA also applies to diagnosis, whereas the ASAS classification criteria can only be used once a diagnosis is made of axSpA or pSpA.

Although intended to classify all forms of SpA at an early stage, the clinical and epidemiological research in axSpA and pSpA has proceeded at a different pace. This may be due to more homogeneous clinical characteristics and an unmistakably added value of imaging in patients classified as axSpA compared with pSpA. Indeed, the axSpA classification criteria recognise two well-defined disease entities, that is, non-radiographic (nr-axSpA) and radiographic axSpA (r-axSpa), marked by the respective absence or presence of radiographic sacroiliitis.^7^ In contrast, the nomenclature of pSpA continues to be more ambiguous. The term *peripheral SpA* has been used interchangeably with some of its subsets such as PsA, ReA and undifferentiated SpA. Moreover, although being the hallmark of pSpA, peripheral symptoms are not pathognomonic as they equally occur in patients classified as axSpA. This considerable overlap has not been acknowledged by the binary ASAS classification system.

### Epidemiology

SpA has a prevalence of 0.9–1.7%,^8 9^ with methodological differences partially accounting for the wide range of estimates across different studies. Importantly, few epidemiological studies used the ASAS classification criteria to define SpA subgroups. Although crude prevalence and incidence rates of pSpA are lacking, the relative prevalence was found to be similar in a Dutch SpA cohort (26.8%),^6^ the Spanish Esperanza cohort (22.8%)^10^ and the Belgian Be-Giant cohort (28.5%).^5^ An unbiased data-driven approach in patients classified as axSpA acknowledged the fact that this group actually consists of two separate patient groups: those with and without peripheral manifestations.^11^ A recent meta-analysis reported pooled prevalence rates of arthritis, enthesitis and dactylitis of 22.9%, 13.6% and 5.6%, respectively, in AS patients. Similar rates were found in nr-axSpA.^12^ The few available data in pSpA suggest a high rate of arthritis (96–98%) compared with enthesitis (41–48%) and dactylitis (40–49%).^5 13^

### Clinical presentation

Similar to the lack of epidemiological information on pSpA, the data on its clinical presentation—other than those extrapolated from PsA studies—are scarce. Compared with axSpA, patients with pSpA are generally older at disease onset. The diagnostic delay is significantly shorter, because pSpA patients usually present with clinically objective signs of inflammation (ie, arthritis or dactylits). In contrast to AS, pSpA shows an equal sex distribution.^6 10^ Typical pSpA manifestations are asymmetrical oligoarthritis of the large joints of the lower limbs, heel enthesitis and dactylitis, the latter being a hallmark of PsA.^14^ Psoriasis is the leading EMM (43–53%) in pSpA, followed by IBD (4–17%) and AAU (2–6%).^6^

Inflammatory back pain, which is obviously a highly prevalent feature in patients with predominant axSpA, has also been reported by 12.5% of PsA^15^ and up to 21% of pSpA patients.^6^ In the Clinical Remission in Early peripheral SPondyloArthritis trial (CRESPA) trial, including patients with early pSpA, 35% had sacroiliitis on MRI, but only 11.6% reported back pain, pointing towards a relevant proportion of patients with subclinical spinal inflammatory disease.^13^ Inversely, the presence of peripheral manifestations in axSpA patients contributes significantly to the burden of disease.^6^

### Genetic susceptibility and pathophysiology

The prevalence of human leucocyte antigen (HLA)-B27 in predominant pSpA ranges from 27% to 47%.^6 10^ The diagnostic and prognostic value of this risk allele has, however, been poorly studied outside the context of axSpA. One Latin-American study also reported a significant association of SpA with HLA-B15, which was almost exclusively found in patients with peripheral involvement. This needs to be confirmed in a larger number of patients with other ethnical backgrounds.^16^ In addition, genome-wide association studies in pSpA are essentially limited to PsA. For example, HLA-B38 and HLA-B39 were found to be linked to polyarticular disease, while dactylitis occurs more frequently in PsA patients carrying the HLA-B2J allele. PsA also associates with genetic polymorphisms involved in the interleukin (IL)-23 signalling pathway (eg, IL-12β and IL-23-receptor), which drives IL-17 production.^17^ The pivotal role of the IL-23/IL-17-axis in PsA has been proven by the successful therapeutic application of monoclonal antibodies targeting these cytokines. In contrast, IL-23 inhibition failed to achieve the primary endpoints in axSpA trials,^18^ which questions some of the proposed disease models. Indeed, IL-23-driven enthesitis has been postulated to be the culprit of inflammation in SpA,^19^ with IL-23 originating from disrupted barrier integrity in patients with, for example, psoriasis. However, this hypothesis may not apply to non-psoriatic subtypes of pSpA. To make the pathophysiology of SpA even more complex, in contrast to PsA and axSpA, IL-17 inhibitors were found to be ineffective in patients with Crohn disease.

## DIAGNOSIS

No global diagnostic algorithm for pSpA has been developed to date; the diagnosis is essentially clinical. The diagnostic work-up starts with an extensive personal and family history to identify concept-related comorbidities (psoriasis, IBD and AAU), (history of) inflammatory back pain, preceding infections and the presence of SpA-related conditions in first- or second-degree relatives. A full system review may reveal clinical clues of other inflammatory or mechanical causes of arthritis, enthesitis or dactylitis. Clinical examination includes a full joint count, palpation of relevant entheses, assessment of dactylitis and careful inspection for minor psoriatic lesions (eg, psoriasis inversa, nasal cleft and hairline) or nail dystrophy. Additional investigations such as HLA-B27 status and imaging of the SIJ in case of suspected axial involvement may contribute to the diagnosis. Both ultrasound (US) and MRI are able to provide objective evidence of inflammation at entheses, certainly when there is patient–evaluator discordance, since a significant proportion of patients with PsA may have coexisting central sensitisation syndrome, which may bias clinical outcome measures.^20^ In recent years, several enthesitis US scoring systems have been published; however, as each is different by incorporating different US elementary lesions, comparison across studies and the use of US as outcome measurement instrument of enthesitis in multicenter studies are problematic.^21^ In 2018, the OMERACT US enthesitis Working Group produced a final reliable US score and definition of enthesitis in SpA/PsA. The US components included in the final definition were hypoechogenicity, increased thickness at enthesis, erosions and calcifications/enthesophytes and Doppler signal at insertion. Further studies are developed for implementing this score in clinical trials and practice.^22^ MRI is also sensitive for detecting enthesitis, but, in contrast to US, it is the only imaging technique that allows detection of perientheseal osteitis.^23–25^ Recently, the OMERACT MRI in Arthritis Working Group has developed and validated an MRI-scoring system for heel enthesitis in SpA/PsA, which is the first composite MRI enthesitis score focused on the heel region, which can be applied in clinical trials.^26^ Nevertheless, one of the key disadvantages of MRI is the limitation to a single body area for scanning. Whole-body (WB) MRI is currently being advanced as a technique that can image multiple areas of the body in one scan done in <1 h which could be helpful in differentiating patients with polyenthesitis from fibromyalgia. The OMERACT MRI group is developing scoring systems for WB-MRI. It remains to be seen if the specificity of findings at the entheses can be improved.

In some patients, there may be a preceding infection with specific bacteria (ie, *Salmonella*, *Shigella*, *Yersinia*, *Campylobacter* and *Chlamydia*). These patients may also develop axial symptoms, inflammatory back pain and even subsequent sacroiliitis. Depending on the predominant manifestations (ie, peripheral or axial), they would be considered to have pSpA or axSpA.

Other (inflammatory) rheumatic conditions always need to be considered as an alternative explanation for the peripheral symptoms, for example, crystal-induced arthropathy or septic arthritis in case of monarthritis, sarcoidosis or seronegative rheumatoid arthritis in patients with oligoarthritis and erosive osteoarthritis when the distal interphalangeal joints are involved.

## CLASSIFICATION CRITERIA

Classification of SpA has been a major issue given the heterogeneous character of the diseases covered by the SpA concept. The first set of classification criteria that encompassed SpA in general dates back to 1991. The European Spondyloarthritis Study Group (ESSG) developed criteria with a focus on two major SpA symptoms (inflammatory back pain and asymmetrical (oligo)arthritis) and required at least one additional clinical or radiological criterion.^27^ Amor *et al* simultaneously developed a classification system based on a list of suggestive clinical, radiologic and laboratory features.^28^ Opposite to the ESSG criteria, Amor criteria did not require an entry criterion. The classification was based on the contribution of 1 to 3 points of each SpA feature; a score of ≥6 points classified a patient as SpA. Both the ESSG and Amor criteria performed similarly in terms of sensitivity and specificity, but especially the ESSG criteria lacked specificity when applied to patients at an early disease stage.^29^

In 2009, ASAS proposed to separate SpA patients in an axial and peripheral subgroup (table 1). This was prompted by the need to recognise their differing clinical presentation, prognosis and therapeutic approach. The performance of the ASAS classification criteria surpassed that of the ESSG and the Amor criteria both in axSpA (sensitivity 82.9%, specificity 84.4%) and in pSpA (sensitivity 77.8%, specificity 82.9%).^3 4^ Peripheral manifestations are listed in both sets of criteria, which adds to their relevance in virtually all subtypes of SpA.

**Table 1:** ASAS classification criteria for peripheral SpA. Adapted from Rudwaleit et al^4^

Peripheral arthritis and/or enthesitis and/or dactylitis* PLUS		
*≥1 SpA feature* Uveitis Psoriasis Crohn’s/colitis Preceding infection HLA-B27 Sacroiliitis on imaging	**OR**	*≥2 other SpA features* Arthritis Enthesitis Dactylitis Inflammatory back pain (in the past) Family history of SpA

*Current peripheral arthritis (compatible with SpA), enthesitis and/or dactylitis, diagnosed clinically by a doctor.

It should be emphasised that, despite the fact that different classification items may provide a framework that helps with the diagnosis of individual patients, they should not be used as diagnostic criteria in order to avoid misdiagnosis and subsequent futile treatments.

## TREATMENT

In the last two decades, the search for new treatment modalities in SpA mainly focused on either axSpA or PsA, considered as the prototype of pSpA. Randomised controlled phase III trials led to the worldwide approval of several biological treatments for these indications. In contrast, all biological treatments are off-label for patients with non-psoriatic pSpA. Although ASAS-EULAR recommendations for axSpA management include recommendations for management of peripheral manifestations^30^ and EULAR and GRAPPA recently updated specific recommendations for PsA management,^31 32^ no specific treatment recommendations for the entity pSpA itself have been published, confirming its neglected status.

### Outcome assessments

To date, no composite measures or response criteria have been identified for use in patients with pSpA. As a consequence, the few randomised controlled trials (RCTs) that have been conducted in pSpA used differing primary endpoints, often borrowed from other diseases such as AS and rheumatoid arthritis (RA). In the ABILITY-2 trial, the first RCT including patients with pSpA fulfilling the current ASAS classification criteria, a new response criterion for pSpA was introduced: the ‘Peripheral SpA 40% Response Criterion’ (PSpARC40). This was defined as ≥40% improvement from baseline (≥20 mm absolute improvement), respectively, in the Visual Analogue Scale (VAS) scores for patient global assessment (PGA) of disease activity and PGA of pain on a 100 mm VAS and ≥40% improvement in at least one of the following scores: (1) 76 swollen joint count and 78 tender joint count; (2) total enthesitis count or (3) dactylitis count.^33^ It was found that this newly developed PSpARC demonstrated a good discriminatory capacity in pSpA, as well as the RA-specific ACR response criteria, the axSpA-specific ASDAS-CRP and BASDAI, and the PGA and physician’s global assessment.^34^ Nevertheless, classical response criteria that are based on the decrease in the number of active joints/entheses are probably not the best evaluation method to assess efficacy in pauci-articular disease. An outcome based on the actual disease activity status would yield more information compared with a mere percentage of improvement. It was in this perspective that in the recent CRESPA trial a more stringent clinical remission criterion as outcome measure was used, defined as the complete absence of arthritis, enthesitis and dactylitis on clinical examination.^13^ Very recently, an ASAS-endorsed international cross-sectional study, the ASAS-PERSPA study was initiated with the objective to measure the prevalence of peripheral involvement in patients with SpA (axSpA, PsA and pSpA) and to evaluate the performance of the current outcome measures in pSpA with the purpose to propose new specific outcome measures for pSpA. This study is now finished, and the results are expected shortly.

### Treatment strategies

The treatment of SpA patients is extremely challenging because of the heterogeneous character of the subsets. Not only is there a different therapeutic approach depending on whether the main presenting rheumatic manifestation is back pain, arthritis, enthesitis or dactylitis, but also the presence and the extent of EMMs significantly influence the therapeutic decisions in an individual patient.

In 2016, both EULAR and GRAPPA published updated recommendations for the management of PsA,^31 32^ including the use of biologics. The GRAPPA recommendation grid provides evidence-based treatment choices for the different domains of psoriatic disease, including typical peripheral manifestations such as arthritis, enthesitis and dactylitis. The EULAR management recommendations for PsA have a single flow chart that focuses on peripheral (poly)arthritis, divided into phases with a sequential approach. Therefore, they suggest the order in which drugs should be prescribed. In patients with active disease despite non-steroidal anti-inflammatory drugs (NSAIDs) and local injections, a classical step-up treatment schedule is proposed with conventional synthetic disease-modifying antirheumatic drugs (csDMARDs), followed—if necessary—by a biological DMARD (bDMARD), such as a tumour necrosis factor (TNF)α inhibitor or a biological targeting IL-12/-23 or IL-17, or a targeted synthetic DMARD (tsDMARD).

Although it seems clinically reasonable to apply these PsA recommendations to other forms of non-psoriatic pSpA, there are only a few RCTs, all involving TNFα inhibitors,^13 33 35 36^ that provide some evidence although insufficient for regulatory approval. As a consequence, the use of TNFα inhibitors in solitary pSpA is considered ‘off-label’ by regulatory agencies worldwide, unless patients also have active axSpA, psoriasis or active IBD, which are among the FDA- and EMA-approved indications for these agents. These regulatory factors clearly limit the management of patients with pSpA.

### Symptomatic treatment: non-steroidal anti-inflammatory drugs and glucocorticoids

NSAIDs are widely used in daily clinical practice for the initial treatment of any type of arthritis. However, controlled studies assessing their efficacy in peripheral arthritis are limited to the field of PsA, with some studies showing good efficacy.^37 38^ NSAIDs are also recommended as the initial treatment choice in patients with peripheral enthesitis or dactylitis, despite the absence of specific studies.^30–32^

Efficacy and side effects of oral or parenteral glucocorticoids have not been studied systematically in pSpA. However, data from the ASAS-ComoSpA cohort suggest that (low-dose) systemic and intra-articular glucocorticoids, especially for monarthritis and oligoarthritis, are quite frequently used in daily practice.^39^ In patients with recent-onset oligoarthritis, an early intervention using intra-articular gluccortocoids followed by sulfasalazine therapy if resistant, reduced synovitis 12 months after treatment compared with those initially treated with only NSAIDs.^40^ Local peritendinous glucocorticoid injections may benefit patients with enthesitis (eg, at the greater trochanter or the plantar fascia), but only a few studies have evaluated its efficacy.^41^

### Conventional synthetic disease-modifying anti-rheumatic drugs (csDMARD)

In patients with persistently active disease because of an inadequate response to the initial therapy, a csDMARD, such as sulfasalazine (2 to 3 g daily), methotrexate (MTX, up to 25 mg once weekly) or leflunomide (20 mg daily), can be initiated.^31 32^ Again, no RCTs were performed in non-psoriatic pSpA. Support for the use of MTX and leflunomide in pSpA is provided by indirect evidence in PsA,^42^ whereas the use of sulfasalazine is indirectly covered by evidence of benefit in AS patients with ReA and peripheral arthritis.^43 44^

MTX is widely used to treat arthritis in PsA, although the first large RCT found no evidence for arthritis improvement.^45^ Interestingly, a subanalysis comparing polyarticular and oligo-articular patients showed a good separation in response between MTX and placebo with regard to the swollen and tender joint count for the polyarticular group but even worsening of the swollen joint count for both the controls and the MTX group in oligo-articular patients. The RESPOND study, an open-label comparison of MTX and infliximab vs MTX monotherapy in early PsA patients, showed superiority of infliximab plus MTX compared with MTX alone, but high MTX response rates were noted (ACR20 66.7% at week 16).^46^ The SEAM study, a double-blind comparison of MTX monotherapy, etanercept monotherapy and combo MTX/etanercept, confirmed superiority of TNFα inhibitors over MTX but also showed marked improvements in arthritis, psoriasis, enthesitis and dactylitis in those receiving only MTX.^47^

In a systematic review of dactylitis associated with PsA, csDMARDs were found to be ineffective.^48^ csDMARDs were also not efficacious for peripheral enthesitis^31 32^ and are therefore not recommended.

### Biological disease-modifying antirheumatic drugs (bDMARDs)

Contrary to csDMARDs, there is convincing evidence for the efficacy of TNFα-blocking agents in patients with pSpA manifestations. They have been successfully evaluated in multiple phase III studies in polyarticular forms of PsA, leading to worldwide approval. TNFα blockade also has a proven beneficial effect on the peripheral manifestations of AS.^49^ A few open-label studies suggested a good efficacy of TNFα blockade in non-AS and non-PsA pSpA.^50–53^ In addition, two RCTs with adalimumab in rather longstanding non-psoriatic pSpA confirmed these findings.^33 35^ Paramarta *et al*^35^ evaluated the efficacy of adalimumab in 40 patients with active pSpA fulfilling the ESSG criteria. At week 12, a clear improvement (based on PGA of disease activity) was observed in the adalimumab group (−31.0± SD 23.3 mm) compared with the placebo group (−5.9± SD 21.4 mm). In the ABILITY-2 study, efficacy and safety of adalimumab were evaluated in 165 pSpA patients.^33^ At week 12, a greater proportion of patients receiving adalimumab achieved the PSpARC40 response compared with patients receiving placebo (39% vs 20%; P=0.006). While the above two studies were performed in longstanding disease (mean disease duration of approximately 7 years), a more recent study, the CRESPA trial, investigated the effect of golimumab in very early forms of pSpA (≤12- week symptom duration). The percentage of patients reaching clinical remission, defined as complete absence of peripheral arthritis, enthesitis and dactylitis on clinical examination, was remarkably high in the golimumab group compared with placebo (75% vs 20%, respectively) at week 24.^13^ Despite the fact that the included study populations in the above-mentioned trials were not exactly identical, a trend was observed towards numerically better efficacy outcomes in patients with shorter symptom duration compared with a more longstanding disease (table 2). While the concept of early treatment is established in other forms of inflammatory arthritis, these data are the first to suggest a similar trend in pSpA. The Paramarta and CRESPA trial also included a withdrawal strategy, evaluating the possibility of drug-free remission. In patients with longstanding disease, discontinuation of TNFα blockade after 12 or 24 weeks resulted in a relapse in 73% of patients within 16 weeks (mean of 10 weeks).^54^ In contrast, the CRESPA trial showed that drug-free remission is an achievable target in early pSpA in at least 50% of patients.^55^

**Table 2:** Comparing study features of anti-TNF trials in pSpA

	CRESPA trial^13 55^	ABILITY-2 trial^33^	Paramarta *et al*^35^ ^54^
**Inclusion criteria**	ASAS classification criteria + rheumatologist diagnosis	ASAS classification criteria + no prior psoriasis, PsA or AS	ESSG or Amor criteria + no AS or PsA
**Multicenter study**	No	Yes	No
**Anti-TNF-blocking agent**	Golimumab	Adalimumab	Adalimumab
**Number of included patients**	60	165	40
**Symptom duration (mean ± SD years)**	5.2 weeks ±2.8 vs 4.4 weeks ±2.0	6.6 years ±6.3 vs 7.7 years ±7.9	7.9 years ±9.3 vs 6.7 years ±6.2
**Primary end point** **% patients in clinical remission at w24** % **patient PSpARC40 response at w12** **PGA of disease activity**	Absence of arthritis, dactylitis and enthesitis at w24 75% vs 20% (p<0.001) 57.5% vs 20% (p=0.0069) −50.0 vs −20.0 (p=0.0015)	PSpARC40 response criteria at w12 ND 39% vs 20% (p=0.006) −27.5 vs −16.4 (p=0.003)	PGA of disease activity ND ND −31.0 vs −5.9 (p=0.001)
**Withdrawal strategy** **Relapse rate after discontinuation**	Yes 47%	No	Yes 73%

AS, ankylosing spondylitis; ASAS, Assessment of Spondyloarthritis International Society; ESSG criteria, European Spondylitis Study Group criteria; PSpARC40 response criteria, peripheral spondyloarthritis 40% response criteria.

Few studies have evaluated the effect of TNFα blockade on solitary peripheral enthesitis: an RCT in patients with NSAID-refractory and MRI-proven persistent heel enthesitis comparing etanercept versus placebo confirmed a significantly greater improvement in disease activity and local heel pain with TNFα blockade.^36^ Of interest, in a prospective randomised controlled open-label study, patients with PsA with active enthesitis were randomised 1:1 to receive either ustekinumab or TNFi.^56^ The primary endpoint was complete clearance of enthesitis, defined by Spondyloarthritis Research Consortium of Canada (SPARCC) index equal to zero at 24 weeks. It was shown that p40-IL-12/IL-23 inhibition was superior to TNFi in the clearance of enthesitis. Future stratified therapeutic approaches in SpA patients may, therefore, consider the presence or absence of enthesitis as a discriminator of response between different cytokine-blocking modalities. A similar observation with regard to enthesitis index was also observed in the SPIRIT-H2H study that compared IL17 inhibition with ixekizumab to adalimumab: a significantly higher proportion of patients reached a SPARCC enthesitis score equal to zero at week 24 in the ixekizumab group.^57^

Data from studies with IL-17 inhibitors demonstrated a beneficial effect comparable with TNFα blockers with regard to arthritis and dactylitis in patients with polyarticular PsA.^58^ Several other biological therapies targeting the IL-23 pathway and small molecules interfering with the phosphodiesterase (PDE)-4 and JAK/STAT pathways have been successfully investigated in polyarticular forms of PsA but not in other forms of pSpA^59–68^ (table 3).

**Table 3:** Evidence-based efficacy of biological and targeted synthetic DMARDs in different SpA manifestations

	AS/axSpA	(polyarticular) PsA	pSpA
TNFi	Yes	Yes	Yes
IL-17i	Yes	Yes	?
IL-12/23i	No	Yes	?
JAKi	Yes	Yes	?
PDE4i	No	Yes	?

AS, ankylosing spondylitis; axSpA, axial spondyloarthritis; DMARDs, disease-modifying antirheumatic drugs; IL-17i, interleukin-17 inhibitor; IL-12/23i, interleukin-12/23 inhibitor; JAKi, Janus kinase inhibitor; PDE4i, phosphodiesterase-4 inhibitor; pSpA, peripheral spondyloarthritis; PsA, psoriatic arthritis; SpA, spondyloarthritis; TNFi, tumour necrosis factor inhibitor.

## FUTURE RESEARCH

Having discussed epidemiology, diagnostic issues, outcome measures and (the lack of evidence-based) treatment options, it is clear that a lot of work still needs to be done in several areas of pSpA, a hitherto neglected entity.

First, the true prevalence of pSpA has not been well studied, and so far there are also insufficient data about the impact of this predominantly oligoarticular disease. Given the fact that it has been shown that the burden of disease in oligo- and polyarticular PsA patients is comparable in terms of quality of life,^69^ a comprehensive health-economic evaluation of pSpA may become increasingly important to justify the use of expensive new treatment options.

Second, the performance of the different outcome measures reflecting disease activity and clinical response in pSpA is unknown. The success of future therapeutic trials depends not only on a well-defined patient population, but also on the availability of valid outcome measures and response criteria. To fully capture typical pSpA manifestations such as arthritis, enthesitis and dactylitis, it may be worthwhile to develop new, pSpA-specific composite measures and response criteria. Currently, the usefulness of the disease-specific PSpARC criteria should be further explored to evaluate if they represent the multiple facets of pSpA disease (face validity), include patient’s and physician’s assessments (face validity) and perform well in RCTs (discrimination).

Third, in contrast to other diseases belonging to the SpA spectrum, there is still a large unmet need to demonstrate the comparable efficacy of csDMARDs, bDMARDs and tsDMARDs in well-designed, randomised trials. This information will be crucial to develop evidence-based recommendations about treatment choices and strategies, such as treat-to-target and early remission induction with the possibility of subsequent longlasting, drug-free remission.
